# Double burden of malnutrition and its associated factors among women in low and middle income countries: findings from 52 nationally representative data

**DOI:** 10.1186/s12889-023-16045-4

**Published:** 2023-08-03

**Authors:** Adugnaw Zeleke Alem, Yigizie Yeshaw, Alemneh Mekuriaw Liyew, Zemenu Tadesse Tessema, Misganaw Gebrie Worku, Getayeneh Antehunegn Tesema, Tesfa Sewunet Alamneh, Achamyeleh Birhanu Teshale, Dagmawi Chilot, Hiwotie Getaneh Ayalew

**Affiliations:** 1 Department of Epidemiology and Biostatistics, Institute of Public Health, College of Medicine and Health Sciences, University of Gondar, Gondar, Ethiopia; 2 Department of Physiology, School of Medicine, College of Medicine and Health Sciences, University of Gondar, Gondar, Ethiopia; 3 Department of Human Anatomy, College of Medicine and Health Science, School of Medicine, University of Gondar, Gondar, Ethiopia; 4 College of Health Sciences, Center for Innovative Drug Development and Therapeutic Trials for Africa (CDT-Africa), Addis Ababa University, Addis Ababa, Ethiopia; 5 Department of Midwifery, School of Nursing and Midwifery, College of Medicine and Health Sciences, Wollo University, Dessie, Ethiopia

**Keywords:** Double burden of malnutrition, Low- and middle-income countries, Women of reproductive age

## Abstract

**Background:**

Double burden of malnutrition (DBM) is an emerging global public health problem. The United Nations member states adopted eradicating all forms of malnutrition as an integral component of the global agenda. However, there is evidence of a high burden of undernutrition among women and rising rates of overweight and obesity, especially in low and middle income countries (LMICs). Therefore, this study aimed to investigate the prevalence and associated factors of underweight, overweight, and obesity among women of reproductive age in LMICs.

**Methods:**

Data for the study were drawn from a recent 52 Demographic and Health Surveys (DHS) conducted in LMICS. We included a sample of 1,099,187 women of reproductive age. A multilevel multinomial logistic regression model was used to identify factors associated with DBM. Adjusted relative risk ratio (RRR) with a 95% Confidence Interval (CI) was reported to show an association.

**Results:**

The prevalence of underweight, overweight, and obesity in LMICs among women of reproductive age was 15.2% (95% CI: 15.1–15.3), 19.0% (95% CI: 18.9- 19.1), and 9.1% (95% CI: 9.0–9.2), respectively. This study found that women aged 24–34 years, aged ≥ 35 years, with primary, secondary, and above educational level, from wealthy households, using modern contraceptives, exposed to media (radio and television), and with high parity (more than one birth) were more likely to have overweight and obesity and less likely to have underweight. Moreover, the risk of having obesity (*RRR* = 0.59; 95% CI = 0.58–0.60 and overweight (*RRR* = 0.78; 95% CI = 0.77–0.79) were lower among rural women, while the risk of being underweight was (*RRR* = 1.13; 95% CI = 1.11–1.15) higher among rural women compared to urban women.

**Conclusion:**

The prevalence of underweight, overweight, and obesity was high among women of reproductive age in LMICs. Underweight, overweight, and obesity are influenced by sociodemographic, socioeconomic, and behavioral-related factors. This study shows that, in order to achieve Sustainable Development Goal 2, a multifaceted intervention approach should be considered to prevent both forms of malnutrition in women of reproductive age. This can be achieved by raising awareness and promoting healthy behaviors such as healthy eating and physical activity, especially among educated women, women from wealthy households, and women exposed to the media.

**Supplementary Information:**

The online version contains supplementary material available at 10.1186/s12889-023-16045-4.

## Background

The double burden of malnutrition (DBM) continues to be a major global public health problem. It is defined as the coexistence of both undernutrition and overnutrition in the same population across the life course [1, 2]. Globally, nearly one-third of the population suffered from at least one form of malnutrition [3]. The double burden of malnutrition is increasing globally, particularly in low and middle income countries (LMICs). Globally, obesity has doubled over the past 30 years, while obesity in LMICs has tripled over the past 20 years [4, 5].

Even though underweight among women has been a major public health concern in LMICs for several decades, due to population aging and increased prevalence of risk factors such as unhealthy diets, physical inactivity, and substance use such as alcohol consumption and cigarette smoking led to a significant shift in epidemiological trend from underweight to overweight and nutritional transitions [6–8]. Nutrition-related diseases and conditions such as nutritional deficiencies, obesity, hypertension, cardiovascular diseases, cancer, and diabetes mellitus are emerging at a faster rate in LMICs than in high-income countries [9]. Overweight/obesity is a major risk for non-communicable diseases (NCDs) morbidity and mortality such as cardiovascular diseases (CVDs), chronic kidney diseases, cancer, musculoskeletal disorders, type 2 diabetes mellitus, and respiratory problems [10–16]. Globally, NCDs are the leading causes of mortality and morbidity, and one of the major challenges of the 21^st^ century [17]. Non-communicable diseases kill 41 million people annually, accounting for 71% of all deaths [18]. Eighty percent of NCD deaths occur in LMICs [19]. The World Health Organization (WHO) projects that by 2030, NCDs will overtake infectious, maternal, neonatal, and nutritional conditions as the leading cause of morbidity and mortality and that the most percentage increase in deaths from NCD will occur in LMICs [20]. Moreover, individuals with underweight are at a major risk of experiencing CVDs including stroke, heart attack, coronary artery disease, and infectious diseases [21].

The DBM is devastating and higher among women than men [4, 5]. It affects their health and the health of their offspring. Overweight/obesity among women is associated with increased pregnancy and childbirth related complications such as gestational diabetes, pre-eclampsia, gestational hypertension, postpartum hemorrhage, instrumental delivery, cesarean delivery, low birth weight, preterm birth, congenital malformation, large-for-gestational-age babies and perinatal death [22–28]. In addition, underweight women are more likely to have pregnancy and childbirth-related complications, such as low birth weight, small for gestational age, preterm birth, and neonatal mortality [22–24, 29, 30].

Although the global prevalence of underweight among women declined from 14.6% in 1995 to 9.7% in 2014, underweight in South Asia and central and East Africa remains unacceptably high and the rate of reduction in underweight is significantly different from country to country [31, 32]. In addition, the global prevalence of obesity among women increased from 6·4% to 14·9% over the past four decades [32]. The prevalence of overweight/obesity among women of reproductive age was 14.9% in Ethiopia, 57.4% in Uganda, 66.7% in Nigeria, 74.1% in Tanzania, 87% in South Africa, 32% in Bangladesh, and 63% in Maldives [33–36].

Studies have assessed factors associated with DBM including age [34, 36–42], educational status [37, 39–45], household wealth status [40, 44–49], breastfeeding [42], marital status [50, 51], place of residence [36, 37, 40, 41, 44], family size [52], types/frequency of diet consumption [51, 52], parity [53], using contraceptives [54, 55], mass media exposure (frequency of watching television, frequency of listing to the radio) [38, 43, 56, 57], and physical activity [51].

The United Nations Sustainable Development Goal 2 (SDG-2) aims to eradicate all forms of malnutrition by 2030 [58]. However, according to the NCD Risk Factors collaborators, there is a zero chance of this being achieved at the global level [59]. Evidence suggests that no country has reversed the rise in obesity at the national level [60]. Also, according to 29 Demographic and Health Survey data and 4 national Surveys, obesity declined only marginally in rural Benin and stabilized in urban areas, and the annual rate of increase in the prevalence of overweight among women of reproductive age in Mexico slowed [61]. Furthermore, previous studies conducted on the prevalence and associated factors of DBM in LMICS were mainly country-specific. Therefore, this study aimed to investigate the prevalence and factors associated with DBM among women of reproductive age in LMICs using nationally representative data. A comprehensive assessment of DBM and its associated factors in at risk populations is critical for developing policies and plans to end all forms of malnutrition and promote well-being by 2030.

## Methods

### Sources of data and sampling procedure

This study used the most recent Demographic Health Survey (DHS) data from 52 LMICs carried out from 2010 to 2021. The DHS is a nationally representative, cross-sectional survey conducted in LMICs that provides reliable data on women, men, and children. The DHS surveys uses uniform data collection procedures, sampling, questionnaires, and coding. This makes the results comparable across countries.

**Fig. 1:**
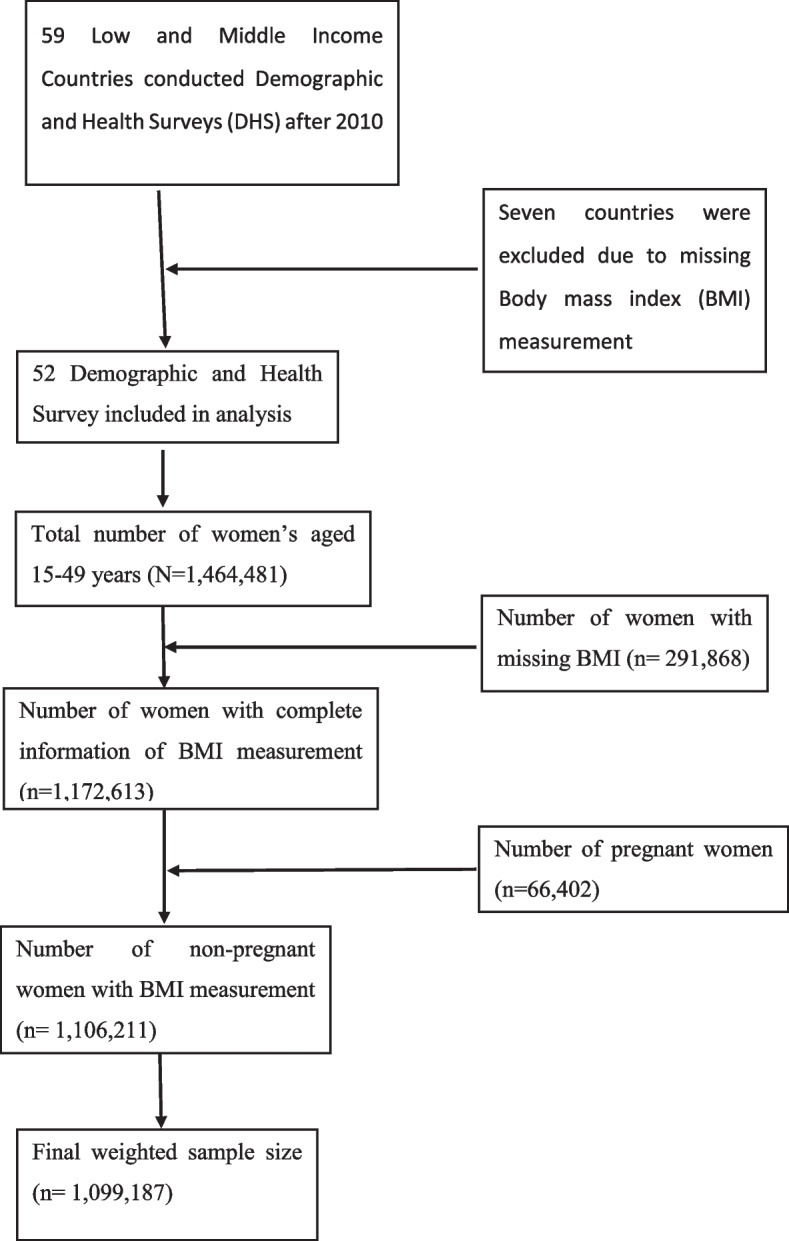
Flow chart for data extraction

To assure the national representativeness, the survey used a two-stage cluster sampling technique. In the first stage, the selection of proportional clusters/enumeration areas was performed using each country’s most recent population and housing census as a sampling frame. In the second stage, a systematic selection of households from the newly created cluster was performed. A detailed description of the DHS sampling design and data collection procedures has been found in each country’s DHS report. A total of 1,464,481 women of reproductive age (15–49 years) were interviewed in 52 LMICs. For this study, a total of 1,099,187 non-pregnant women of reproductive age who had a body mass index (BMI) measurement were used for analysis (Fig. 1).

### Study variables and measurement

#### Outcome variable

Body mass index derived from women’s weight in kilograms divided by the square of her height in meters (kg/m^2^) was the dependent variable. The weight and height were measured using standard technique by trained field technicians. Electronic Seca scales with a digital screen were used to measure weight and a stadiometer were used measure height [62]. According to WHO cutoff points, BMI was divided into four categories as underweight if the BMI is < 18.5 kg/m^2^, normal if it is 18.5–24.9 kg/m^2^, overweight if it is 25–29.9 kg/m^2^ and obese if it is ≥ 30 kg/m^2^ [63].

#### Independent variables

Based on previous literatures [37, 38, 40, 41, 49, 53, 64, 65], several independent variables such as age, educational status of women, mass media access (frequency of watching television, frequency of listening to the radio, and frequency of reading newspaper/magazines), accessing health care, working status, birth order, terminated pregnancy, household wealth status, family size, sex of household head, marital status, parity, and contraceptive use were included as the individual level variables of the study. Whereas residence was considered as the community level variable in this study. Detailed coding and operation definitions of variables are presented in Supplementary Table 1.

#### Data processing and analyses

Datasets were appended together to explore the pooled prevalence of underweight, overweight, obesity, and its associated factors among women of reproductive age in LMICs. Data cleaning and statistical analysis were carried out using STATA version 16 [66]. All statistical analysis was performed based on sample weighting. Frequencies and percentages were used to describe the background characteristics of the study participants. To identify associated factors of underweight, overweight, and obesity we used the multilevel multinomial logistic regression since BMI (dependent variable) is a categorical variable with four categories and DHS data are hierarchical, i.e. individuals were nested within communities. Normal BMI was used as the reference group. In particular, four models were constructed; null model contains only the outcome variable and clusters to assess the random effects between clusters, model I contains individual-level variables only, model II includes a community-level variable only, and model III includes both individual and community-level variables. The best-fitted model was selected by using deviance to identify factors associated with DBM and the model with the least deviance was selected (model III).

The Intra-class Correlation Coefficient (ICC), and the Median Odds Ratio (MOR) were computed to assess the clustering effect/variability. The intra-class correlation was calculated for each of the models as ICC = the variance of each model/ (variance of each model + 3:29) [67] and the MOR was calculated as; MOR = exp(0.95√ cluster level variance) [68]. Proportional Change in Variance (PCV) was computed for models I, II, and III with respect to the variance in the empty model as PCV = (variance of the empty model—variance of the model with more terms (model I, II, or III) / variance of the empty model [68].

First, we fitted a bivariable multilevel multinomial logistic regression model for each independent variable to select variables for multivariable analysis, and variables with *p*-value ≤ 0.20 in the bivariable multilevel multinomial logistic regression analysis were included in multivariable analysis (Supplementary Table 2). Finally, results for the multivariable analysis have been presented as adjusted relative risk ratio (RRR), with their corresponding 95% confidence intervals (CI).

## Results

### Background characteristics of study participants

A total of 1,099,187 women in LMICs were included in the study. Of the total, 876,682 (79.8%) were from male-headed households, 554,009 (50.8) watched television at least once a week, 599,994 (54.6%) did not use contraceptives and 756,027 (68.8%) were currently in union. Nearly two-thirds (705,926, 64.2%) of study participants were rural residents, and 495,824 (45.1%) women had secondary education. The mean age of the participants was 30.0 ± 9.9 years (Table 1).

**Table 1:** Background characteristics of women of reproductive age in LMICs, 2010–2021

Variables	Frequency	Percent
Age		
Mean ± SD	30.3 ± 9.9	
15–24	370,336	33.7
25–34	332,638	30.3
35–49	396,213	36.0
Educational status		
Not educated	247,490	22.5
Primary	212,586	19.3
Secondary	495,824	45.1
Higher	143,287	13.1
Household wealth status		
Poorest	198,179	18.0
Poorer	215,199	19.6
Middle	224,562	20.4
Richer	231,009	21.0
Richest	230,238	21.0
Marital status		
Currently in union	756,027	68.8
Not currently in union	343,160	31.2
Working status		
Not working	287,813	54.5
Working	240,128	45.5
Family size		
≤ 5	416,937	37.9
6–10	616,785	56.1
> 10	65,465	6.0
Frequency of reading newspaper or magazine		
Not at all	737,359	67.6
Less than once a week	193,452	17.7
At least once a week	158,576	14.5
Almost every day	1,426	0.2
Frequency of watching television		
Not at all	337,284	30.9
Less than once a week	190,570	17.5
At least once a week	554,009	50.8
Almost every day	8,898	0.8
Frequency of listening to radio		
Not at all	768,762	70.5
Less than once a week	139,771	12.8
At least once a week	175,588	16.1
Almost every day	6,748	0.6
Sex of household head		
Male	876,682	79.8
Female	222,505	20.2
Residence		
Urban	393,261	35.8
Rural	705,926	64.2
Ever had terminated pregnancy		
No	951,168	86.6
Yes	147,989	13.4
Contraceptive use		
Not using	599,994	54.6
Use traditional method	423,756	38.5
Use modern method	75,429	6.9
Currently breastfeeding		
No	902,426	82.1
Yes	196,761	17.9
Parity		
Nulipara	318,835	29.0
Primiparous	152,514	13.9
Multiparous	506,966	46.1
Grand Multiparous	120,872	11.0
Accessing health care		
Not big problem	568,872	52.6
Big problem	512,150	44.4

### Prevalence of underweight, overweight and obesity

**Fig. 2:**
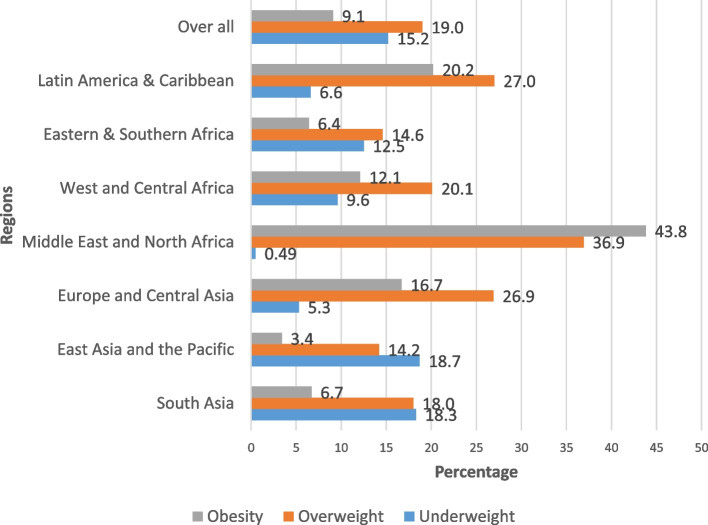
Regional prevalence of underweight, overweight and obesity among women of reproductive age

The pooled prevalence of underweight in LMICs among women of reproductive age was 15.2 (95% CI: 15.1–15.3), ranging from 0.2% in Egypt to 26.3% in Timor-Leste. The pooled prevalence of overweight in LMICs among women of reproductive age was 19.0 (95% CI: 18.9–19.1), ranging from 5.9% in Ethiopia to 37.8% in Jordan. The pooled prevalence of obesity in LMICs among women of reproductive age was 9.1 (95% CI: 9.0–9.2) (Fig. 2) and it ranged from 1.6% in Ethiopia to 47.8% in Egypt (Table 2).

**Table 2:** Prevalence of underweight, overweight and obesity by countries among women of reproductive age

World region	Countries	Survey year	Underweight Frequency (%)	Overweight Frequency (%)	Obesity Frequency (%)
**South Asia**	Maldives	2016/17	713(10.5)	2,031(30.0)	1,320(19.5)
	Nepal	2016	1,057(17.2)	1,04717.0)	313(5.1)
	Bangladesh	2017/18	2,207( 11.8)	4,781(25.6)	1,218 (6.5)
	India	2019/21	124,181(18.6)	117,188(17.6)	43,215(6.5)
	Pakistan	2017	336(8.7)	1,172(30.3)	831(21.4)
	Pooled prevalence	2016–2021	128,494(18.3)	126,219(18.0)	46,897(6.7)
**East Asia and the Pacific**	Cambodia	2014	1,501(13.9)	1,637(15.2	301(2.8)
	Myanmar	2014/15	1.880(15.4)	2,329(19.1)	678(5.7)
	Timor-Leste	2016	3,102(26.3)	969(8.2)	194(1.6)
	**Pooled prevalence**	2014–2016	6,483(18.7)	4,935(14.2)	1,173(3.4)
**Europe and Central Asia**	Albania	2017/18	460(4.4)	3,006(28.8)	1,733(16.6)
	Armenia	2015/16	207(3.6)	1,712(30.0)	858(15.0)
	Tajikistan	2017	718(7.3)	2,351(23.8)	317(13.3)
	Kyrgyz Republic	2012	540(7.2)	1,790(23.8)	897(11.9)
	Turkey	2013	290(3.5)	2,365(28.7)	2,188(26.6)
	**Pooled prevalence**	2012–2018	2,215(5.3)	11,224(26.9)	5,993(16.7)
**Middle East and North Africa**	Egypt	2014	46(0.2)	7,106(36.5)	9,305(47.8)
	Jordan	2017/18	80(1.2)	2,433(37.8)	2.020(31.4)
	**Pooled prevalence**	2014–2018	126(0.49)	9,539(36.9)	11,325(43.8)
**West and Central Africa**	Burkina Faso	2010	192(15.5)	612(7.9)	293(3.8)
	Benin	2017/18	781(10.8)	1,184(16.5)	667(9.3)
	Central Democratic Congo	2013/14	1,167(14.1)	1,036(12.5)	281(3.4)
	Cote d’vore	2011/12	319(7.6	790(18.8)	291(6.9)
	Chad	2014	1,860(18.9)	885(9.0)	239(2.4)
	Cameroon	2018	380(6.1)	1,478(23.6)	843(13.4)
	Congo	2011/12	705(13.6)	866(16.7	626(12.1)
	Mauritania	2019/21	524(7.7)	1,837(26.9)	1,832(26.8)
	Gabon	2012	350(7.0)	1,252(25.1)	1,018(20.4)
	Ghana	2014	265(6.1)	1,081(24.8)	661(15.2)
	Gambia	2019/20	740(13.4)	1,229(22.3)	747(13.9)
	Guinea	2018	455(9.4)	873(17.3)	412(8.5)
	Liberia	2019/20	202(5.3)	919(24.2)	464(12.2)
	Mali	2018	459(10.1)	862(19.0)	384(8.5)
	Nigeria	2018	1,564(11.9)	2,389(18.1)	1,305(9.9)
	Niger	2011	661(14.4)	603(13.1)	375(8.1)
	Sierra leone	2019	476(6.7)	1,397(19.7)	565(8.0)
	Senegal	2012	1,158(20.2)	822(14.3)	748(13.0)
	Togo	2013/14	303(6.9)	855(19.5)	486(11.1)
	**Pooled prevalence**	2010–2021	12,561(9.6)	20,970(20.1)	12,237(12.1)
**Eastern & Southern Africa**	Burundi	2016/17	1,482(18.7)	491(6.2)	167(1.7)
	Ethiopia	2016	3,093(22.0)	835(5.9)	223(1.6)
	Comoros	2012	333(6.7)	1,196(24.0)	730(14.7)
	Kenya	2014	1,181(8.8)	3,033(22.7)	1,363(10.2)
	Lesotho	2014	135(4.2)	809(25.2)	626(19.5)
	Madagascar	2021	1,627(18.3)	985(11.1)	244(2.7)
	Malawi	2015/16	522(7.1)	1,124(15.2)	416(5.6)
	Mozambique	2011	1,024(8.4)	1,480(12.1)	616(5.0)
	Namibia	2013	552(13.8)	734(18.4)	530(13.2)
	Rwanda	2019/20	399(5.8)	1,410(20.6)	397(5.8)
	South Africa	2016	96(3.1)	834(26.6)	1,118(35.7)
	Tanzania	2015	1,117(9.3)	3,102(18.4)	1,206(10.0)
	Uganda	2016	463(8.6)	896(16.6)	383(7.1)
	Zimbabwe	2015	545(6.00)	2,032(22.4)	1,141(12.6)
	**Pooled prevalence**	2011–2021	12,569(12.5)	18,961(14.6)	9,160(6.4)
**Latin America & Caribbean**	Dominican Republic	2013	625(7.2)	2,578(29.8)	1,793(20.7)
	Guatemala	2014/15	688(2.8)	7,720 (31.9)	4,814(19.9)
	Haiti	2016/17	954(10.5)	1,865(20.6)	1,017(11.2)
	Honduras	2011/12	1,012(4.7)	6,154(28.6)	5,115(23.7)
	**Pooled prevalence**	2011–2017	3,279(6.6)	18,317(27.0)	12,739(20.2)

### Multilevel analyses

#### Random parameter estimation and model selection

Table 3 shows the multilevel multinomial regression model of random effects estimates of DBM. Random effect analysis in the null model was used to test for clustering effects on DBM. The results showed a significant difference in DBM between clusters (ICC = 9.9%), which indicated that the clusters accounted for 9.9% of the variance in DBM. In Model III, variability in DBM between clusters was reduced (ICC, 7.3%). In the model I and model III, the explained variances were 16.7% and 27.8% respectively. This implied that a large amount of variances in DBM has been explained by model III. To identify factors associated with DBM, model III which contains both individual and community level variables was selected as the most suitable due to the least deviance. Therefore, the final interpretation of results (fixed results) was based on model III. Fixed effect results of model I and model 2 are presented in Supplementary Table 3.

**Table 3:** The random effects of the multilevel multinomial logistic regression in assessing the factors associated with DBM

Parameters	Null model	Model I	Model III	Model III
Community level variance	0.36	0.30	0.34	0.26
Intraclass Correlation Coefficient (ICC)	0.099	0.083	0.094	0.073
Proportional Change in Variance (PCV)	Reference	0.167	0.056	0.278
Median Odds Ratio (MOR)	1.77	1.68	1.74	1.62
Deviance (-2Log-likelihood)	2,456,811.4	2,244,835.0	242,176.8	2,239,708.2

Null model contains only the outcome variable and cluster numbers, model I; a model that includes only individual-level variables, model II, a model that includes only community-level variables, and model III; a model that includes both individual and community-level variables

#### Factors associated with underweight, overweight and obesity (Fixed effects)

Table 4 presents the estimated adjusted relative risk ratios (RRR) with their 95% CIs from multi-level multinomial logit models on underweight, overweight, and obesity for women of reproductive age in LMICs. Women aged 25–34 years and ≥ 35 years were 1.94 (*RRR* = 1.94, 95% 1.90–1.97) and 2.69 (*RRR* = 2.69, 95% 2.64–2.75) times higher risks for having overweight compared to women aged 15–24 years, respectively. Similarly, women aged 25–34 years and ≥ 35 years were 2.48 (*RRR* = 2.48, 95% 2.41–2.55 and 4.18 (*RRR* = 4.18, 95% 4.05–4.31) times higher risks for obesity compared to women aged 15–24 years, respectively. Conversely, women in the age group 25–34 years had 36% (*RRR* = 0.64, 95% 0.63–0.66) and ≥ 35 years had 47% (*RRR* = 0.53, 95%CI; 0.52–0.52) lower risks for having underweight compared to women aged 15–24 years. Women who belonged to households of the poorer (*RRR* = 1.24; 95% CI = 1.22–1.27), middle (*RRR* = 1.48, 95% CI = 1.46–1.51), richer (*RRR* = 1.72, 95% CI = 1.68–1.75), and richest (*RRR* = 1.98; 95% CI = 1.94–2.02) wealth quintiles had a higher risk of experiencing the overweight compared to women from poorest households. Also, women who belonged to households of the poorer (*RRR* = 1.13; 95% CI = 1.10–1.17), middle (*RRR* = 1.41, 95% CI = 1.37–1.45), richer (*RRR* = 1.72, 95% CI = 1.67–1.77), and richest (*RRR* = 2.20; 95% CI = 2.14–2.27) wealth quintiles had significantly higher risks of experiencing the obesity relative to women from poorest households. While women from the richest, richer, middle, and poorer households had 43% (*RRR* = 0.57, 95%CI; 0.55–0.56), 33% (*RRR* = 0.67, 95%CI; 0.66–0.68), 26% (*RRR* = 0.74, 95%CI; 0.73–0.75), and 17% (*RRR* = 0.83, 95%CI; 0.81–0.84) times lower risk of having underweight compared to women from poorest households, respectively. Being rural dwellers was associated with a 1.13 (*RRR* = 1.13; 95% CI = 1.11–1.15) times higher risk of having underweight, but at decreased risk of overweight and obesity by 22% (*RRR* = 0.78; 95% CI = 0.77–0.79) and 41% (*RRR* = 0.59; 95% CI = 0.58–0.60) respectively compared to urban dwellers.

**Table 4:** Multivariable multi-level multinomial analysis of the factors associated with malnutrition in LMICs

Variables	Underweight ARRR (95% CI)	Overweight ARRR (95% CI)	Obesity ARRR (95% CI)
Age			
15–24	1	1	1
25–34	0.64 (0.63–0.66)**	1.94 (1.90–1.97) **	2.48 (2.41–2.55) **
35–49	0.53 (0.52–0.54)**	2.69 (2.64–2.75) **	4.18 (4.05–4.31) **
Educational status			
Not educated	1	1	1
Primary	0.74 (0.73–0.75)**	1.33 (1.31–1.35) **	1.54 (1.50–1.57) **
Secondary	0.86 (0.85–0.88)**	1.32 (1.31–1.35) **	1.48 (1.44–1.51) **
Higher	0.68 (0.66–0.69)**	1.40 (1.37–1.44) **	1.39 (1.35–1.44) **
Household wealth status			
Poorest	1	1	1
Poorer	0.83 (0.81–0.84) **	1.24 (1.22–1.27) **	1.13 (1.10–1.17) **
Middle	0.74 (0.73–0.75)**	1.48 (1.46–1.51) **	1.41 (1.37–1.45) **
Richer	0.67 (0.66–0.68)**	1.72 (1.68–1.75) **	1.72 (1.67–1.77) **
Richest	0.57 (0.55–0.59)**	1.98 (1.94–2.02) **	2.20 (2.14–2.27) **
Marital status			
Not currently in union	1	1	1
Currently in union	1.01 (0.97–1.03)	1.04 (0.99–1.05)	1.02 (0.98–1.04)
Family size			
≤ 5	1	1	1
6–10	1.12 (1.11–1.14)**	0.97 (0.94–1.03)	0.98 (0.96–1.02)
> 10	1.08 (1.05–1.11)**	0.96 (0.93–1.01)	1.02 (0.99–1.06)
Frequency of reading newspaper or magazine			
Not at all	1	1	1
Less than once a week	1.05 (1.00–1.08)	1.00 (0.99–1.02)	0.99 (0.97–1.01)
At least once a week	1.03 (0.98–1.06)	1.04 (1.02–1.06) *	1.05 (0.99–1.08)
Almost every day	0.98 (0.76–1.15)	1.03 (0.88–1.21)	1.13 (0.93–1.37)
Frequency of watching television			
Not at all	1	1	1
Less than once a week	1.02 (0.99–1.04)	1.06 (0.96–1.08)	1.04 (0.9–1.11)
At least once a week	1.01 (0.99–1.03)	1.52 (1.50–1.54) **	2.25 (2.20–2.30) **
Almost every day	0.78 (0.71–0.86)*	1.63 (1.52–1.74) **	2.81 (2.59–3.05) **
Frequency of listening to radio			
Not at all	1	1	1
Less than once a week	0.94 (0.91–1.01)	1.01 (0.97–1.05)	1.10 (0.97–1.19)
At least once a week	0.58 (0.57–0.59)**	1.34 (1.32–1.36) *	1.77 (1.73–1.80) **
Almost every day	0.57 (0.52–0.63)**	1.23 (1.14–1.32) *	1.62 (1.48–1.77) **
Sex of household head			
Male	1		
Female	0.96 (0.95–1.09)	1.03 (0.96–1.05)	1.05 (0.99–1.10)
Residence			
Urban	1	1	1
Rural	1.13 (1.11–1.15)**	0.78 (0.77–0.79) **	0.59 (0.58–0.60) **
Contraceptive use			
Not using	1	1	1
Use traditional method	0.96 (0.93–1.02)	1.09 (0.95–1.11)**	0.97 (0.89–1.12)**
Use modern method	0.92 (0.90–0.93)*	1.00 (0.98–1.01)	1.15 (1.12–1.17) **
Currently breastfeeding			
No	1	1	1
Yes	0.97 (0.92–1.03)	0.78 (0.77–0.79)**	0.69 (0.68–0.71)**
Parity			
Nulliparous	1	1	1
Primiparous	0.61 (0.59–0.62)**	1.75 (1.71–1.79) **	1.99 (1.93–2.06) **
Multiparous	0.64 (0.62–0.65) **	1.91 (1.87–1.95) **	2.47 (2.39–2.54) **
Grand Multiparous	0.67 (0.65–0.69) **	1.92 (1.87–1.97) **	3.16 (3.04–3.28) **

*ARRR* Adjusted relative risk ratio, *CI* Confidence interval

^*^P-value < 0.05, **P-value < 0.01

The risk of being overweight was higher among women with primary education (*RRR* = 1.33; 95% CI = 1.31–1.35), secondary education (*RRR* = 1.32, 95%CI = 1.31–1.35), and higher education (*RRR* = 1.40, 95%CI = 1.37–1.44) compared with non-educated women. The risk of obesity was higher among women with primary education (*RRR* = 1.54; 95% CI = 1.50–1.57), secondary education (*RRR* = 1.48, 95%CI = 1.44–1.51), and higher education (*RRR* = 1.39, 95%CI = 1.35–1.44) compared women without formal education. However, compared with women without formal education, women with primary education had 26% (*RRR* = 0.74; 95% CI = 0.73–0.75), secondary education had 14% (*RRR* = 0.86, 95%CI = 0.85–0.88) and higher education had 32% (*RRR* = 0.68, 95%CI = 0.66–0.69) lower risk of underweight. Respondents living with a family size of 6–10 had 12% (*RRR* = 1.12, 95%CI = 1.11–1.14) and > 10 had 8% (*RRR* = 1.08, 95%CI = 1.05–1.11) higher risk of underweight compared to women living with ≤ 5 family size.

The risk of being overweight was higher among primiparous (*RRR* = 1.75, 95%CI = 1.71–1.79), multiparous (*RRR* = 1.91, 95%CI = 1.87–1.95), grand multiparous women (*RRR* = 1.92, 95%CI = 1.87–1.97), women who watched television at least once a week (*RRR* = 1.52, 95%CI = 1.50–1.54), almost every day (*RRR* = 1.63, 95%CI = 1.52–1.74), women who listened to the radio at least once a week (*RRR* = 1.34, 95%CI = 1.32–1.36), and almost every day (*RRR* = 1.23, 95%CI = 1.14–1.32).

The risk of obesity was higher among primiparous (*RRR* = 1.99, 95%CI = 1.93–2.06), multiparous (*RRR* = 2.47, 95%CI = 2.39–2.54), grand multiparous (*RRR* = 3.16, 95%CI = 3.04–3.28), women who watched television at least once a week (*RRR* = 2.25, 95%CI = 2.20–2.30), and almost every day (*RRR* = 2.81, 95%CI = 2.59–3.05), women who listened to the radio at least once a week (*RRR* = 1.77, 95%CI = 1.73–1.80), and almost every day (*RRR* = 1.62, 95%CI = 1.48–1.77). Lactating mothers were less likely to be overweight (*RRR* = 0.78, 95%CI = 0.77–0.79) and obese (*RRR* = 0.69, 95%CI = 0.68–0.71) compared to non-lactating mothers. Moreover, the risk of underweight was less likely among primiparous (*RRR* = 0.61, 95%CI = 0.59–0.62), multiparous (*RRR* = 0.64, 95%CI = 0.62–0.64), grand multiparous (*RRR* = 0.67, 95%CI = 0.65–0.69), those using modern contraceptives (*RRR* = 0.92, 95%CI = 0.90–0.93), those watching television almost every day (*RRR* = 0.78, 95%CI = 0.71–0.81), those listening radio at least once a week (*RRR* = 0.58, 95%CI = 0.57–0.59), and almost every day (RRR = 0.57, 95%CI = 0.52–0.63)compared to counterparts (Table 4).

## Discussion

This study examined the prevalence and associated factors of DBM indicators (underweight, overweight, and obesity) among women of reproductive age in LMICs using 52 nationally representative data. This study builds literature on sociodemographic, socioeconomic, obstetric*,* and behavioral factors associated with DBM among women of reproductive age in LMICs based on a nationally representative survey. The results indicated a substantial DBM among women of reproductive age in LMICs. We found that educational status, age, household wealth status, frequency of watching television, frequency of radio listening, parity, use of modern contraceptives, not lactating and urban dwelling were positively associated with overweight and obesity. Educational status, age, household wealth status, frequency of watching television, frequency of radio listening, parity, use of modern contraceptives, family size and urban dwelling were negatively associated with being underweight. The findings of this study will assist policymakers to identify the population groups at risk of DBM for better development of programs, which could in turn play a substantial role in reducing the burden of non-communicable diseases.

In the present study, the pooled prevalence of underweight is 15.2 (95% CI: 15.1, 15.3). Furthermore, we found that a high burden of being overweight (nearly one in five women) and obese (nearly one in 10 women) co-occurs with a high burden of being underweight in LMICs. Prevalence rates for underweight, overweight, and obesity show considerable variation across countries. Variations were found in other studies as well [69, 70]. This difference may actually be due to differences in physical activity levels, dietary habits and awareness of malnutrition in different countries. Previous studies indicated that the prevalence of obesity was more than 20% in 14 Latin American countries [70] and more than 30% in several countries in the Middle East and in North and southern Africa among women [59]. A previous study reported overweight and obesity in LMICs ranged from a low of 4.7% in the Democratic Republic of Korea to a high of 88.3% in Tonga [70]. The contribution of underweight, overweight, and obesity to the burden of disease and mortality have been well documented [71–73]. The existing evidence shows that underweight and obesity are among the top 10 leading risk factors for the global burden of disease. Decreased physical activity and** c**hanges in diet are among the main contributors to obesity [74, 75]. In addition to undernutrition, a profound shift in nutrition from the end of famine (pattern 3) to the consumption of more energy-dense diets (pattern 4) is a public health concern for most LMICs and requires urgent action. This shift from a traditional diet to a Western-style diet is a key factor contributing to the prevalence of obesity-related NCDs in LMICs [76–78]. In response to the rising prevalence of DBM, WHO has introduced a comprehensive strategy to stop the increase in obesity prevalence by 2025, with the potential to simultaneously reduce the risk of undernutrition and diet-related NCDs [79, 80]. This comprehensive strategy is designed to provide easy access to a healthy and nutritious diet that promotes a healthy weight. Specifically, the WHO proposed a roadmap so-called Double-Duty Actions (DDAs) to tackle the DBM. This road map includes programs, policies, and interventions that have the potential to simultaneously reduce the risk or burden of all forms of malnutrition [81]. Public health actions such as dietary/nutrition counseling, media outreach, nutrition labeling, issuing of dietary guidelines, and taxes on sugar-sweetened beverages are widely recommended as part of national strategies to combat overweight, obesity, and obesity-related NCDs [82]. However, such public health actions are not common in most LMICs [78]. In addition, interventions to address overweight/obesity should focus on women of reproductive age to increase their awareness of the impact of healthy foods such as vegetables, grains, and fruits, as well as television viewing and a sedentary lifestyle. Therefore, we strongly recommend DDAs activities such as adopting healthy dietary habits during adolescence and antenatal care nutritional counseling should be strengthened to reduce both forms of malnutrition among women of reproductive age.

This study found that a positive relationship exists between the frequency of watching television overweight and obesity. This finding was consistent with previously published studies from Ghana [43], Tanzania [38], Bangladesh [56], and Myanmar [57] that showed women who watched television had a higher risk of being overweight and obese compared to those who did not watch television. This result is not surprising given that a positive association between spending a long time watching television and an increase in sitting time has been shown, which results in a reduced level of physical activity and reduced resting metabolism [56, 83, 84]. A study revealed that the effects of television watching extend beyond reduced levels of physical activity to increased calorie consumption and influence people to make unhealthy diet choices while watching as a result of advertising [85–87]. A Study conducted in Ghana and Kenya documented nearly half (48.3%) of all advertisements were for sugar-sweetened beverages [85]. Moreover, in LMICs, having a television is a proxy indicator for higher socioeconomic status, which in turn increases the consumption of energy-dense and junk foods [38].

This study showed that women from the richest, richer and middle households were more likely to be overweight and obese while less likely to be underweight compared to those who resided in the poorest households. Likewise, previous studies have consistently shown that women from wealthy households have a higher risk of being overweight and obese, and a lower risk of being underweight [40, 46–49]. In LMICs, unhealthy practices such as consuming more energy-dense diets and following a sedentary lifestyle vary by socioeconomic status. Furthermore, women living in the poorest households are less likely to have access to adequate and diverse diets, water, clothing, and good shelter. As a result, the poorest people are at high risk of developing from various communicable diseases due to macronutrient or micronutrient deficiency, poor hygiene, and sanitation [88, 89].

Global evidence shows that education is one of the most important media that influence the economy, attitude, health behaviors, and outcomes, including physical activity, diet, and body weight [90–92]. Thus, educated individuals have better health status, due to the improvement in socioeconomic status, health information, and health behaviors. However, this was not the case in this study, which suggests that women of childbearing age with primary, secondary, and tertiary education were more likely to be overweight and obese compared to women with no formal education. This finding is similar to previous studies conducted in LMICs [37, 40–43]. A possible explanation for this, in LMICs, higher educational attainment is associated with higher socioeconomic status and material resources which in turn women to more likely take Western diets, which are characterized by high protein and energy-dense foods, and use a vehicle for transport or practice more sedentary employment (for example, office work) [93]. However, in contrast to our study, a study from china found that women of reproductive age with secondary and higher education were less likely to be overweight and obese and not significantly associated with being underweight [39]. This inconsistency may be attributable to differences in socioeconomic, policy, and nutritional transition across population groups. The association between education and overweight/obesity is contextual, varies over time, and is closely related to the ongoing nutritional transition across countries [94, 95]. Furthermore, this disparity may also suggest that health policies in LMICs have traditionally focused on health problems related to infectious diseases and undernutrition, while interventions targeting rapid shifts in diet and epidemiology are likely underway. Therefore, we hypothesize that establishing a causal relationship between educational status and DBM indicators is difficult because of possible confounding with unobserved characteristics. Future researchers should address the causal relationship between education and obesity by conducting experimental or quasi-experimental studies.

The results in relation to age were mixed. While in general a significant positive association was observed between age and overweight and obesity, a negative association was observed with underweight. These findings are consistent with previous studies. A study by Amugsi DA et al., found that older age was positively associated with being overweight and obese in Ghana, Mozambique, Kenya, and Nigeria [42]. In the same study, older age was positively associated with being overweight in Democratic Republic of Congo. Moreover, older age was associated positively with overweight among women in Ethiopia, Tanzania, Maldives, Bangladesh, India, and China [34, 37–41]. Consistent with our findings, studies from Ethiopia, Nigeria, and India found that older age was negatively associated with being underweight [36, 41, 42]. In contrast, Amugsi DA et al., and Song J et al., reported that older women were more like to be underweight [39, 42]. The positive relationship found between older age and overweight and obesity may potentially be explained by several factors. First, advanced age is associated with parity and another associated factor of overweight and obesity. Evidence documented that women usually gain weight during pregnancy which could be associated with higher lifetime weight retention if weight loss does not occur post-partum [96–99]. As observed in this study, primiparous, multiparous, and grand multiparous women were more likely to be overweight and obese and less likely to be underweight as compared with nulliparous women. Second, body composition and hormonal changes that occur during aging may contribute to fat accumulation [100]. Third, the fact that adolescence is a time of rapid physical, psychosocial, and cognitive development increases the need for nutrients that may be linked to undernutrition. The relationship between age and underweight has been documented in previous studies. For example, a study in Ethiopia and India observed a significant negative association between age and underweight [36, 41].

Consistent with other studies in the LMIC region [36, 37, 40, 41], we found that compared with rural women, urban women were more at risk of being overweight and obese, while urban women were less likely to be underweight. Similarly, a study in china demonstrated that urban women were more likely to be overweight and obese [39]. Possible reasons for the association between urban dwellers and overweight and obesity may be that urban dwellers are more likely to consume processed, packaged and refrigerated foods, and to be physically inactive. Besides, women who resided in rural areas may be engaged in occupational physical activities such as agricultural occupations subject them to labor-intensive activities and therefore, unlikely to gain as much weight as urban women could be a possible explanation for the negative relationship between rural residents and overweight and obesity.

Furthermore, this study revealed that the risk of being underweight is lower among modern contraceptive users. Previous research from Nigeria and Myanmar has observed similar relationships [54, 55]. Also, we found the risk of obesity is higher among modern contraceptive users. This result is supported by a study among women of reproductive age in Ethiopia; it revealed that using combined oral contraceptives increased the occurrence of obesity [101]. Additionally, similar findings have been reported in studies conducted in Kenya, Myanmar and India [41, 101, 102]. The reason behind this association may be the consequence of might be hormonal effect of contraceptives (i.e. progesterone and estrogen) that contribute to weight gain. Progesterone increases appetite and estrogen facilitates lipid metabolism and fat accumulation which in turn increases obesity [103].

We found that risk of being overweight and obese was less likely among lactating mothers. Consistent with our findings, Amugsi DA et al. suggested that the risk of being overweight and obese in the five countries was less likely among lactating mothers [42]. Besides, health benefits of breastfeeding for both mothers and babies have been documented [104–109]. Breastfeeding provides a child with ideal nutrition, protects child from certain diseases, and supports growth and development [104, 105]. Breastfeeding can also help to reduce breast and ovarian cancer, type 2 diabetes, postpartum depression, and high blood pressure [106–109].

### Strengths and limitations of study

This study has its own strengths and limitations. The main strength of this study is the use of large nationally representative samples with appropriate statistical modeling. The use of large nationally representative data and multilevel analysis helps to provide more robust estimates of observed associations as well as enhance the generalizability of the results. Although this study used a nationally representative dataset and appropriate model, the results should be interpreted in light of some limitations. First, this study used cross-sectional data, which does not provide itself to the establishment of a temporal relationship between the factors and outcome variables. Second, we are unable to incorporate important covariates such as physical activity, dietary intake, other comorbid conditions, and energy expenditure, as the DHS did not collect information on these variables. Third, although BMI is important WHO recommended indicator of nutritional status measurement, it cannot differentiate between body fat and lean body mass. Furthermore, this study did not examine DBM at the population and within households can be considered as a limitation of the study.

## Conclusion

The prevalence of underweight, overweight, and obesity was high among women of reproductive age in LMICs. Educational status, age, household wealth status, frequency of watching television, frequency of listening to radio, parity, and using modern contraceptives were positively associated with overweight and obesity and negatively associated with being underweight. Moreover, being rural dweller and breastfeeding were negatively associated with obesity and overweight, and rural women had a higher risk of being underweight than urban women. This study shows that in order to achieve Sustainable Development Goal 2, multifaceted intervention approaches should be considered to prevent both forms of malnutrition in women of reproductive age. This can be achieved by raising awareness and promoting healthy behaviors such as healthy eating and physical activity, especially among educated women, women from wealthy households, and women exposed to the media.

### Supplementary Information

**Additional file 1: Supplementary Table 1.** Coding strategy of variables used in analysis.

**Additional file 2: Supplementary Table 2.** Bivariable multi-level multinomial analysis of the factors associated with double burden of malnutrition in low and middle income countries.

**Additional file 3: Supplementary Table 3.** Fixed effects of multi-level multinomial analysis of individual and community level variables.

## Data Availability

The datasets used and/or analysed during the current study is available in a public, open access repository which is accessible online www.measuredhs.com.

## Abbreviations

CI: Confidence Interval
DBM: Double Burden of Malnutrition
DHS: Demographic and Health Survey
RRR: Relative risk ratio
LMICs: Low and Middle income Countries
