# Supplementary material for: Double burden of malnutrition and its associated factors among women in low and middle income countries: findings from 52 nationally representative data
Source: BMC Public Health. 2023 Aug 3;23:1479. doi: 10.1186/s12889-023-16045-4 (PMC10398981; doi:10.1186/s12889-023-16045-4)
Supplement: Supplementary file 1 — Additional file 1: Supplementary Table 1. Coding strategy of variables used in analysis. [file 12889_2023_16045_MOESM1_ESM.docx]

**Supplementary Table 1**: Coding strategy of variables used in analysis

| Variables | Categorization / operationalization |
| --- | --- |
| Body mass index (BMI) | Women having BMI 18.5-24.9 kg/m^2^ were coded as “0”normal weight, < 18.5 kg/m^2^ were coded as “1” underweight, 25-29.9 kg/m^2^ were coded as “2”overweight, and ≥ 30 kg/m^2^ were coded “3”obese |
| Age | The age of women’s was categorized as 15-24 years “1”, 25-34 years “2”, and 35-49 years “1” |
| Educational level | The educational status of women was categorized as not educated “1”, primary “2”, secondary “3” and higher “4” |
| Mothers marital status | The marital status of the women was categorized as not currently in union “1” or currently in union “2”. |
| Household wealth status | The wealth index was categorized as poorest “1”, poorer “2” middle “3”, richer “4”, and richest “5” |
| Frequency of watching television | It was categorized as not at all “1”, less than once a week “2”, at least once a week “3”, and almost every day “4” |
| Frequency of listening to the radio | Frequency of listening to the radio was categorized as not at all “1”, less than once a week “2”, at least once a week “3”, and almost every day “4” |
| Frequency of reading newspaper/magazines | It was categorized as not at all “1”, less than once a week “2”, at least once a week “3”, and almost every day “4” |
| Accessing health care | Accessing health care was generated from women’s perception on getting the money needed for treatment (big problem/not a big problem), distance to a healthcare facility (big problem/not a big problem), getting medical help for self: getting permission to go (big problem/not a big problem) and getting medical help for self: wanting to go alone (big problem/not a big problem). Then coded as a big problem “1” if a woman faces at least one problem and no a big problem “2” if a women reports none of the above problems |
| Family size | Number of household members were recoded as 1 “if number of household members is ≤5”, 2 if number of household members is 6-10”, and 3 “ if number of household members is >10” |
| Sex of household head | Categorized as Male “1” and Female “2” |
| Contraceptive use | It was categorized as not using any contraceptive methods “1”, using traditional methods “2” ,and using modern contraceptive methods “3” |
| Residence | It was grouped as urban “1” and rural “2” |
| Parity | Parity status of women was categorized as nuliparaous (no birth history) “1, primiparous (one birth) “2” , multiparous (2-4 births) “3”, and grand multiparous (≥5 births) |
| Ever had a terminated pregnancy | It was categorized as no “1” and yes “2” |

Note: BMI; Body mass index, kg/m^2^; Kilogram per square meter
