# Supplementary material for: Double burden of malnutrition and its associated factors among women in low and middle income countries: findings from 52 nationally representative data
Source: BMC Public Health. 2023 Aug 3;23:1479. doi: 10.1186/s12889-023-16045-4 (PMC10398981; doi:10.1186/s12889-023-16045-4)
Supplement: Supplementary file 2 — Additional file 2: Supplementary Table 2. Bivariable multi-level multinomial analysis of the factors associated with double burden of malnutrition in low and middle income countries. [file 12889_2023_16045_MOESM2_ESM.docx]

**Supplementary Table 2:** Bivariable multi-level multinomial analysis of the factors associated with double burden of malnutrition in low and middle income countries

| Variables | Underweight | | Overweight | | Obesity | |
| --- | --- | --- | --- | --- | --- | --- |
|  | CRRR (95% CI) | P-Value | CRRR (95% CI) | P-Value | CRRR (95% CI) | P-Value |
| Age |  |  |  |  |  |  |
| 15-24 | 1 | 1 | 1 | 1 | 1 | 1 |
| 25-34 | 0.51(0.50,0.53) | <0.001 | 2.52(2.48,2.56) | <0.001 | 3.39(3.31,3.47) | <0.001 |
| 35-49 | 0.43(0.41,0.44) | <0.001 | 3.57(3.52,3.62) | <0.001 | 6.19(6.06,6.33) | <0.001 |
| Educational status | |  |  |  |  |  |
| Not educated | 1 | 1 | 1 | 1 | 1 | 1 |
| Primary | 0.79(0.77,0.80) | <0.001 | 1.34(1.32,1.36) | <0.001 | 1.69(1.65,1.73) | <0.001 |
| Secondary | 1.14(1.13,1.16) | 0.03 | 1.24(1.22,1.25) | <0.001 | 1.41(1.38,1.44) | <0.001 |
| Higher | 0.81(0.81,0.83) | <0.001 | 1.55(1.52,1.57) | <0.001 | 1.73(1.69,1.77) | <0.001 |
| Household wealth status | |  |  |  |  |  |
| Poorest | 1 | 1 | 1 | 1 | 1 | 1 |
| Poorer | 0.82(0.81,0.83) | <0.001 | 1.39(1.36,1.41) | <0.001 | 1.35(1.31,1.39) | <0.001 |
| Middle | 0.72(0.71,0.74) | <0.001 | 1.82(1.79,1.86) | 0.02 | 1.98(1.93,2.03) | <0.001 |
| Richer | 0.63(0.60,0.64) | <0.001 | 2.31(2.27,2.35) | <0.001 | 2.86(2.78,2.93) | <0.001 |
| Richest | 0.52(0.50.0.53) | <0.001 | 2.99(2.94,3.04) | <0.001 | 4.45(4.34,4.55) | <0.001 |
| Marital status | |  |  |  |  |  |
| Not currently in union | 1 | 1 | 1 | 1 | 1 | 1 |
| Currently in union | 0.93(0.90,1.02) | 0.08 | 1.08(0.97,1.12) | 0.123 | 1.20(.99,1.23) | 0.06 |
| Family size |  |  |  |  |  |  |
| ≤5 | 1 | 1 | 1 | 1 | 1 | 1 |
| 6-10 | 1.20(1.11,1.24) | 0.04 | 0.83(0.79,0.90) | <0.001 | 0.85(0.81,0.88) | <0.001 |
| >10 | 1.12(1.09,1.20) | <0.001 | 0.79(0.75,0.83) | <0.001 | 0.84(0.81,0.87) | <0.001 |
| Frequency of reading newspaper or magazine | |  |  |  |  |  |
| Not at all | 1 | 1 | 1 | 1 | 1 | 1 |
| Less than once a week | 1.03(0.96,1.08) | 0.132 | 1.08(0.99,1.15) | 0.06 | 1.06(0.99,1.09) | 0.08 |
| At least once a week | 0.95(0.92,1.01) | 0.09 | 1.19(1.12,1.23) | 0.03 | 1.15(1.06,1.20) | 0.04 |
| Almost every day | 0.87(0.82,0.92) | 0.02 | 1.22(1.15,1.24) | <0.001 | 1.18(1.13,1.22) | 0.03 |
| Frequency of watching television | |  |  |  |  |  |
| Not at all | 1 | 1 | 1 | 1 | 1 | 1 |
| Less than once a week | 0.98(0.94,1.04) | 0.112 | 1.06(0.99,1.09) | 0.06 | 1.15(1.08,1.23) | 0.04 |
| At least once a week | 0.92(0.87,0.99) | 0.07 | 2.04(2.02,2.08) | <0.001 | 3.18(3.12,3.24) | <0.001 |
| Almost every day | 0.84(0.81,0.88) | <0.001 | 2.21(2.04,2.38) | <0.001 | 4.98(4.66,5.33) | <0.001 |
| Frequency of listening to radio | |  |  |  |  |  |
| Not at all | 1 | 1 | 1 | 1 | 1 | 1 |
| Less than once a week | 0.95(0.91,1.01) | 0.06 | 1.08(1.04,1.10) | 0.03 | 1.23(1.21,1.27) | <0.001 |
| At least once a week | 0.88(0.84,0.94) | <0.001 | 1.38(1.35,1.40) | <0.001 | 2.01(1.97,2.05) | <0.001 |
| Almost every day | 0.82(0.77,0.88) | <0.001 | 1.31(1.23,1.40) | <0.001 | 2.16(2.00,2.33 | <0.001 |
| sex of household head | |  |  |  |  |  |
| Male | 1 | 1 | 1 | 1 | 1 | 1 |
| Female | 0.93(0.89,1.01) | 0.103 | 1.03(0.97,1.08) | 0.08 | 1.12(1.07,1.14) | 0.03 |
| Residence |  |  |  |  |  |  |
| Urban | 1 | 1 | 1 | 1 | 1 | 1 |
| Rural | 1.32(1.30,1.34) | <0.001 | 0.52(0.51,0.53) | <0.001 | 0.34(0.33,0.35) | <0.001 |
| Contraceptive use | |  |  |  |  |  |
| Not using | 1 | 1 | 1 | 1 | 1 | 1 |
| Use traditional method | 0.80(0.77,0.93) | 0.02 | 1.64(1.59,1.66) | <0.001 | 1.62(1.60,1.66) | <0.001 |
| Use modern method | 0.98(0.95,1.04) | 0.165 | 1.26(1.22,1.31) | <0.001 | 1.05(0.99,1.11) | 0.06 |
| Currently breastfeeding | |  |  |  |  |  |
| No | 1 | 1 | 1 | 1 | 1 | 1 |
| Yes | 0.91(0.87,0.94) | <0.001 | 0.33(0.31,0.36) | <0.001 | 0.53(0.51,0.59) | <0.001 |
| Accessing health care | |  |  |  |  |  |
| Not big problem | 1 | 1 | 1 | 1 | 1 | 1 |
| Big problem | 1.03(0.92,1.12) | 0.245 | 0.98(0.91,1.03) | 0.314 | 0.97(0.93,1.04) | 0.212 |
| Parity |  |  |  |  |  |  |
| Nulliparous | 1 | 1 | 1 | 1 | 1 | 1 |
| Primiparous | 0.52(0.49,0.55) | <0.001 | 2.12(2.08,2.16) | 0.01 | 2.48(2.42,2.55) | <0.001 |
| Multiparous | 0.48(0.46,0.50) | <0.001 | 2.73(2.69,2.77) | <0.001 | 2.71(2.69,2.77) | <0.001 |
| Grand Multiparous | 0.50(0.47,0.52) | <0.001 | 2.29(2.24,2.33) | <0.001 | 3.86(3.76,3.97) | <0.001 |
| Ever had terminated pregnancy | |  |  |  |  |  |
| No | 1 | 1 | 1 | 1 | 1 | 1 |
| Yes | 0.94(0.91,1.01) | 0.298 | 1.06(0.98,1.13) | 0.211 | 1.04(0.97,1.11 | 0.199 |

**Note:** CRRR: crude relative risk ratio, CI: confidence interval
