# Supplementary material for: Double burden of malnutrition and its associated factors among women in low and middle income countries: findings from 52 nationally representative data
Source: BMC Public Health. 2023 Aug 3;23:1479. doi: 10.1186/s12889-023-16045-4 (PMC10398981; doi:10.1186/s12889-023-16045-4)
Supplement: Supplementary file 3 — Additional file 3: Supplementary Table 3. Fixed effects of multi-level multinomial analysis of individual and community level variables. [file 12889_2023_16045_MOESM3_ESM.docx]

**Supplementary Table 3:** Fixed effects of multi-level multinomial analysis of individual and community level variables

| **Model I** (Model with individual level variables) | | | |
| --- | --- | --- | --- |
| Variables | Underweight  RRR (95% CI) | Overweight  RRR (95% CI) | Obesity  RRR (95% CI) |
| Age |  |  |  |
| 15-24 | 1 | 1 | 1 |
| 25-34 | 0.66 (0.64-0.68)** | 1.86 (1.82-1.89) ** | 2.39 (2.31-2.46) ** |
| 35-49 | 0.54 (0.52-0.55)** | 2.59 (2.54-2.64) ** | 4.02 (3.89-4.14) ** |
| Educational status |  |  |  |
| Not educated | 1 | 1 | 1 |
| Primary | 0.73 (0.71-0.75)** | 1.34 (1.32-1.36) ** | 1.55 (1.51-1.59) ** |
| Secondary | 0.85 (0.84-0.87)** | 1.33 (1.31-1.36) ** | 1.49 (1.45-1.52) ** |
| Higher | 0.65 (0.63-0.67)** | 1.41 (1.38-1.44) ** | 1.42 (1.38-1.47) ** |
| Household wealth status |  |  |  |
| Poorest | 1 | 1 | 1 |
| Poorer | 0.82 (0.81-0.84) ** | 1.25 (1.22-1.27) ** | 1.15 (1.11-1.18) ** |
| Middle | 0.72 (0.71-0.74)** | 1.53 (1.50-1.56) ** | 1.52 (1.48-1.57) ** |
| Richer | 0.64 (0.63-0.67)** | 1.86 (1.82-1.89) ** | 2.07 (2.01-2.13) ** |
| Richest | 0.53 (0.51-0.54)** | 2.28 (2.23-2.32) ** | 2.99 (2.91-3.08) ** |
| Marital status |  |  |  |
| Not currently in union | 1 | 1 | 1 |
| Currently in union | 0.98 (0.96-1.02) | 1.13 (1.11-1.16)* | 1.08 (0.98-1.13) |
| Family size |  |  |  |
| ≤5 | 1 | 1 | 1 |
| 6-10 | 1.11 (1.10-1.14)** | 0.94 (0.91-1.01) | 0.96 (0.94-0.98)* |
| >10 | 1.07 (1.04-1.10)** | 0.98 (0.94-1.02) | 1.01 (0.97-1.04) |
| Frequency of reading newspaper or magazine |  |  |  |
| Not at all | 1 | 1 | 1 |
| Less than once a week | 1.07 (1.04-1.09)* | 1.00 (0.99-1.02) | 0.99 (0.97-1.01) |
| At least once a week | 1.02 (1.00-1.05) | 1.05 (1.03-1.07) * | 1.09 (0.99-1.13) |
| Almost every day | 0.91 (0.73-1.12) | 1.06 (0.90-1.24) | 1.17 (0.97-1.41) |
| Frequency of watching television |  |  |  |
| Not at all | 1 | 1 | 1 |
| Less than once a week | 1.02 (0.99-1.04) | 1.16 (1.14-1.19)* | 1.16 (0.99-1.20) |
| At least once a week | 1.01 (0.99-1.03) | 1.54 (1.52-1.56) ** | 2.34 (2.29-2.39) ** |
| Almost every day | 0.78 (0.71-0.86)* | 1.82 (1.70-1.95) ** | 3.47 (3.40-4.76) ** |
| Frequency of listening to radio |  |  |  |
| Not at all | 1 | 1 | 1 |
| Less than once a week | 1.02 (0.99-1.04) | 1.10 (1.08-1.13)* | 1.23 (1.20-1.27)* |
| At least once a week | 1.00 (0.98-1.02) | 1.38 (1.35-1.40) ** | 1.85 (1.81-1.89) ** |
| Almost every day | 0.72 (0.66-0.80)** | 1.24 (1.14-1.33) * | 1.64 (1.50-1.79) ** |
| Sex of household head |  |  |  |
| Male | 1 |  |  |
| Female | 0.92 (0.89-1.01) | 1.11 (1.08-1.15)* | 1.09 (0.99-1.15) |
| Contraceptive use | |  |  |
| Not using | 1 | 1 | 1 |
| Use traditional method | 0.99 (0.97-1.02) | 1.04 (0.94-1.06) | 0.88(0.81-0.91)* |
| Use modern method | 0.94 (0.93-0.96)* | 0.97 (0.95-1.02) | 1.13 (1.09-1.18) ** |
| Currently breastfeeding |  |  |  |
| No | 1 | 1 | 1 |
| Yes | 1.09 (1.07-1.11)* | 0.76 (0.74-0.77)** | 0.67 (0.65-0.69)** |
| Parity |  |  |  |
| Nulliparous | 1 | 1 | 1 |
| Primiparous | 0.68 (0.66-0.71)** | 1.51 (1.47-1.55) ** | 1.78 (1.74-1.84) ** |
| Multiparous | 0.72 (0.70-0.74) ** | 1.64 (1.59-1.69) ** | 2.16 (2.04-2.24) ** |
| Grand Multiparous | 0.76 (0.73-0.79) ** | 1.92 (1.87-1.97) ** | 2.81 (2.70-2.92) ** |
| **Model II** (Model with community level variable) | | | |
| Residence |  |  |  |
| Urban | 1 | 1 | 1 |
| Rural | 1.32 (1.30,1.34)** | 0.52 (0.51,0.53)** | 0.34(0.33,0.35)** |

**Note:** *P-value<0.05, **P-value <0.01, RRR: relative risk ratio, CI: confidence interval
